# Inclusion of Sainfoin in the Diet Might Alter Strongyle Infection in Naturally Infected Horses

**DOI:** 10.3390/ani12080955

**Published:** 2022-04-07

**Authors:** Pauline Grimm, Noémie Laroche, Samy Julliand, Gabriele Sorci

**Affiliations:** 1 Lab To Field, 26 bd Docteur Petitjean, 21000 Dijon, France; noemie.laroche@lab-to-field.com (N.L.); samy.julliand@lab-to-field.com (S.J.); 2 Biogéosciences, CNRS UMR 6282, Université de Bourgogne Franche-Comté, 6 Boulevard Gabriel, 21000 Dijon, France; gabriele.sorci@u-bourgogne.fr

**Keywords:** equine, nematodes, diet, polyphenols, FEC, larval motility

## Abstract

**Simple Summary:**

Control of equine parasites using synthetic drugs raises concerns, as drug resistance is increasing, especially for cyathostomins which are very common parasites in horses. As these parasites can be harmful for equine health, it is therefore necessary to find alternative solutions to control their populations. Sainfoin (*Onobrychis viciifolia*) is a legume that contains high levels of polyphenols, and that has demonstrated anti-parasitic activity in ruminants. This study aimed to assess whether sainfoin could also be a natural solution to regulate equine cyathostomins. We observed that the horses that consumed around 1.7 kg sainfoin per day excreted less parasites eggs in the feces, suggesting that sainfoin either decreases the number of intestinal adult worms or alters their fertility. However, we did not find any effect of the diet on egg excretion following the treatment with an anthelmintic drug. The parasite larvae that developed from eggs coming from horses fed sainfoin were less motile, which can be beneficial to reduce pasture contamination. Further studies are needed to understand the mechanisms that lead to a possible reduction in egg excretion and to provide recommendations for practitioners.

**Abstract:**

It is increasingly difficult to control equine strongyles with synthetic drugs, as resistance is commonly observed. Here, we investigated the possible anthelmintic effect of sainfoin (*Onobrychis viciifolia*), a polyphenol-rich legume, in naturally infected horses. On Day 0 (D0), 17 horses were allocated to three different homogenous groups in terms of fecal egg count (FEC): the control group (CONT) received a diet composed on a DM basis of 83% hay and 17% wheat bran, while in the sainfoin 1 (SF1) and sainfoin 2 (SF2) groups, half or all wheat bran, respectively, was replaced by dehydrated sainfoin pellets. The infection dynamics were monitored by weekly FEC, from D0 to D84. On D28, all horses were treated with fenbendazole. Larval motility was assessed from coprocultures at D0, D28, D56 and D84. Horses in Group SF2 had lower FEC from D7 to D28. After fenbendazole treatment, no effect of the diet was measured on FEC. Both before and after anthelmintic treatment, larvae from horses consuming sainfoin were less motile than larvae from the CONT group. These results suggest that sainfoin has an in vivo anthelmintic activity in naturally infected horses, although this effect appears to be context-dependent.

## 1. Introduction

Controlling gastrointestinal nematode infections is a major challenge for the equine industry, as strongylosis caused by the most prevalent equine parasites (small strongyles or cyathostomins and large strongyles) can result in lethargy, anemia, weight loss, diarrhea, malabsorption, colic or even death [1,2]. Since 1960, synthetic anthelmintic drugs have been frequently and systematically used by veterinary practitioners for both therapeutic and prophylactic purposes. This has inevitably selected for anthelminthic resistance, and, not surprisingly, resistance to drugs is now commonly observed, especially in cyathostomins [3]. Controlling these strongyles with the currently used molecules is therefore more difficult [4]. In addition, although treating equine infections is necessary for animal health, the use of anthelmintic drugs has been associated with an increased risk of developing colic in the week following an anthelminthic treatment [5]. This could result from the dysbiosis incurred in the equine hindgut by anthelminthic treatment, since decreases in both microbiota diversity [6,7] and the concentration of cellulolytic bacteria [8] have been reported following anthelminthic treatment. In addition to the possible negative consequences for the microbiota, fecal anthelminthic residues can affect the microfauna and macrofauna of the surrounding pastures, surface waters and ground waters, which can adversely impact pastural and aquatic biodiversity [9]. Thus, there is a growing need for alternative strategies to control equine nematodes.

Using natural products as dietary supplements might be one of these alternatives to the widespread use of synthetic drugs [3]. Among these natural products, sainfoin *(Onobrychis viciifolia)*, which contains high levels of condensed tannins and other polyphenols [10,11], has already been shown to reduce nematode burden and/or egg shedding in sheep [12,13,14,15,16,17], goats [18,19,20] and, more recently, cattle [21]. In most of these studies, sainfoin was fed as hay, but similar effects have been observed when administered as pellets [15,21], suggesting that processed sainfoin keeps its anthelmintic properties. In heavily infected animals, a rapid reduction in the excretion of parasite eggs has been reported when sainfoin was supplemented in the diet compared to a control diet [13,19]. In non-infected goats and lambs exposed to gastrointestinal nematodes, adding sainfoin to the diet resulted in lower infection rates compared to control groups [16,18]. This feeding strategy has also been shown to improve host resilience in the face of the infection. In previously dewormed goats exposed to naturally infected pastures, sainfoin-fed animals maintained lower excretion of parasite eggs and better health, while poor health and higher mortality rates were reported in goats fed a control diet [18]. However, other in vivo studies have only reported parasite-specific effects (e.g., against *Ostertagia ostertagi* and not against *Cooperia oncophora* [21]), stage-specific effects (reduction of *Haemonchus contortus* adult numbers, reduction of *Cooperia curticei* per capita fecundity [13]), or no effect at all of sainfoin [22,23]. This heterogeneity might be accounted for by different factors, including the feedstuff containing sainfoin (concentrations and biochemical structures of polyphenols, quantity supplied), and the digestive systems of the host species [24].

In horses, to the best of our knowledge, a single study has assessed the anthelminthic effect of pelleted sainfoin, both in vitro and in vivo [25]. In vitro results showed that sainfoin decreased hatching rates and impaired larval development into the infective stage. However, these results were not confirmed in vivo, as the number of eggs excreted in the feces of horses who received a control was similar to the number excreted by those who received a sainfoin diet over the course of 18 days. Similarly, there were no differences between diets in worm counts (based on worms expelled in feces after a deworming treatment), juvenile-to-adult strongyle count ratio or female strongyle fecundity [25]. Further investigations are thus required to evaluate the possible anthelminthic properties of sainfoin and identify the conditions that could potentially lead to sainfoin-associated health improvement in horses.

Here, we report the result of an experiment aiming at assessing the anthelmintic properties of sainfoin in horses naturally infected with strongyles. Animals were fed with two doses of sainfoin (a third group received a control diet) over the course of three months, and we assessed the effect of the diet on strongyle infection before and after an anthelminthic treatment in order to evaluate the effect on reinfection.

## 2. Materials and Methods

### 2.1. Animals, Diets and Experimental Design

Seventeen adult gelding “Trotteur Français” horses (mean ± SD: 7 ± 2 years-old; 526 ± 34 kg) were recruited based on their natural intestinal parasitism (mainly cyathostomins) at the start of the trial (mean ± SD: 738 ± 499 strongyle eggs per gram [EPG] of feces, with a minimum threshold of 100), and included in a longitudinal study of 85 days (D0 to D84) from February to April 2020. During the seven weeks prior the start of the trial, all horses were kept in individual stables with access to a dry paddock but not to pasture. Their last anthelminthic treatment had occurred more than 4 months before the start of the experiment. Horses were housed in 13.5 m^2^ individual boxes bedded with straw. At D0, horses were allocated to the experimental groups to ensure similar initial weight, age and fecal egg count (FEC) among groups (Table 1).

From D1 to D84, horses were fed one of three diets. The CONT group (5 horses) received a control diet composed on a dry matter (DM) basis of 83% meadow hay and 17% pelleted wheat bran. Half (8.5% DM) or full (17% DM) quantities of wheat bran were substituted with dehydrated sainfoin pellets in the diets of Group SF1 (6 horses) and Group SF2 (6 horses), respectively (Table 2). The provided daily amount of dehydrated sainfoin pellets (Perly cultivar, Equifolia^®^, Multifolia, Viapres Le Petit, France) corresponded to 1.53 g DM/kg BW (SF1) or 3.06 g DM/kg BW (SF2). Sainfoin contained 2.6% DM of condensed tannins and 2.7% DM of total phenols (Folin method N-AOEN/M/119, Inovalys Laboratory, Nantes, France); tannins and phenols were considered negligible in hay and wheat bran. The three diets were formulated to meet the energetic requirements for light-exercised horses according to the NRC recommendations [26], and to be iso-DM and almost iso-energy and iso-protein. Daily hay ingestion was fixed at 15 g DM/kg BW, based on the latest European recommendation to ensure equine ethological needs and for health considerations [27]. Rations were provided in two equal meals per day, at 08:00 and 16:45. Hay was provided on the ground, and pellets of sainfoin or wheat bran in a feeder. Horses had free access to water and salt lick blocks (Solsel^®^, K + S Minerals and Agriculture GmbH, Kassel, Germany).

Horses were weighed weekly during the trial. They were all treated on D28 with 7.5 mg of fenbendazole/kg BW (Panacur^®^ oral paste, Intervet, Angers, France). This molecule, selected for its short egg reappearance period, had never been used in our farm. Thus, two different experimental periods were considered: Period 1 (D0 to D28), corresponding to the pre-anthelminthic treatment period, and Period 2 (D35 to D84), corresponding to the post-anthelminthic administration period. Due to climatic reasons, horses had access to paddocks every day for 2 h from D1 to D47, and to pasture for 6 h per day from D48 until the end of the trial. During the grazing period, horses were divided into 6 groups of 2 or 3 animals, which rotated every day on one of the 6 different overgrazed parcels to ensure homogeneous exposure to natural infection.

### 2.2. Parasitism Assessment

Fresh feces were collected in the rectum of each horse before the start of the experimental diets (D0), and then on a weekly basis (from D7 to D84). Individual fecal egg count (FEC) was assessed using a modified McMaster technique [28]. A fecal sample of 3.00 g was stirred in 42 mL of a NaCl solution (300 g/L, specific gravity of 1.2). The obtained suspension was filtered through a sieve (500 µm pore size) and the filtrate was then inserted in the two chambers of a McMaster slide. After ten minutes, the number of eggs was enumerated under a microscope with a detection limit of 50 eggs per gram of feces.

*FEC* at *D*28 (before the anthelmintic treatment), *D*35 (7 days post-treatment) and *D*42 (14 days post-treatment) were used to compute fecal egg count reduction for each horse as follows:

(1) $$FECRT_{D35}=\frac{FEC_{D28}-FEC_{D35}}{FEC_{D28}}\times 100$$

(2) $$FECRT_{D42}=\frac{FEC_{D28}-FEC_{D42}}{FEC_{D28}}\times 100$$

*FECR* was also computed for the whole group of horses to assess the efficacy of fenbendazole [29] as follows:

(3) $$FECR=1-\frac{a}{b}$$

where *a* and *b* are the post- and pre-treatment arithmetic means of *FEC*, respectively. The uncertainty intervals of FECR were computed according to Equations (5) and (6) reported in Levecke et al., 2018 [30].

At D0, D28, D56 and D84 fecal samples were also cultured to allow larval development. For each horse, 20.0 g of feces were spread in a Petri dish, which was placed in a humidity chamber at 25 °C for 15 days. Larvae were extracted from each culture using the Baermann technique, with 20–30 mL of physiological solution (9 g NaCl/L). After 6 h of larval migration, sediments were collected and centrifuged into 15 mL tubes for 10 min at 1500 rpm. After centrifugation, aggregates were collected and diluted in 1 mL of physiological solution. Larvae were observed in 100 µL solution placed on a glass slide under a microscope. Larval motility was assessed on samples that contained at least 3 larvae/100 µL, according to the following scale: 0—all larvae immobile; 1—immobile larvae and low motility larvae; 2—low motility larvae; 3—low motility and vigorous motility larvae; 4—all larvae vigorously motile.

### 2.3. Statistical Analyses

A one-way ANOVA (PROC ANOVA, SAS 9.3) was run on values of body weight, age and FEC at Day 0 to check whether the experimental groups had similar initial values (Table 1). An ANOVA was also run to test if diet had an effect on FECRT.

The effect of diet on changes in logarithmically transformed FEC (log_10_[FEC + 1]) and body weight was assessed using mixed-effect models with a normal distribution of errors (PROC MIXED, SAS 9.3). Two models were run, one for each experimental period (pre- and post-anthelmintic treatment). Day, Diet and the interaction Day × Diet were included as fixed effects, and the Day was included as a repeated measure per horse. In addition to mean values, diet also might have affected the inter-individual variation in FEC. To test this, coefficients of variation (CV) of logarithmically transformed FEC were computed for each Day × Diet combination; these values were used as the dependent variables in a mixed-effect model with a normal distribution of errors, including the same fixed and repeated effects described above (except the Day × Diet interaction that cannot be estimated here). For all significant effects, least-squares means comparison was performed using a Tukey adjustment. Effects of the Day and Diet and their impacts on larval motility scores were analyzed using a mixed-effect model with a multinomial distribution of errors and a Laplace likelihood estimation method (PROC GLIMMIX, SAS 9.3). The correlation between the number of larvae observed and larval motility scores was assessed using a Pearson’s correlation coefficient (PROC CORR, SAS 9.3). For all statistical analyses, the significance threshold was set at *p* < 0.05.

## 3. Results

### 3.1. Zootechnical Measurements

No feed refusals were observed during the trial, and all horses remained healthy. Body weight did not vary during the trial (*p* = 0.99 in Period 1 and *p* = 0.99 in Period 2), nor was it affected by Diet (*p* = 0.79 in Period 1 and *p* = 0.58 in Period 2) or Day × Diet interaction (*p* = 1.00 in Period 1 and *p* = 1.00 in Period 2).

### 3.2. Effect of Diet on Fecal Egg Count during the Pre-Anthelmintic Treatment Period (D0 to D28)

**Figure 1:**
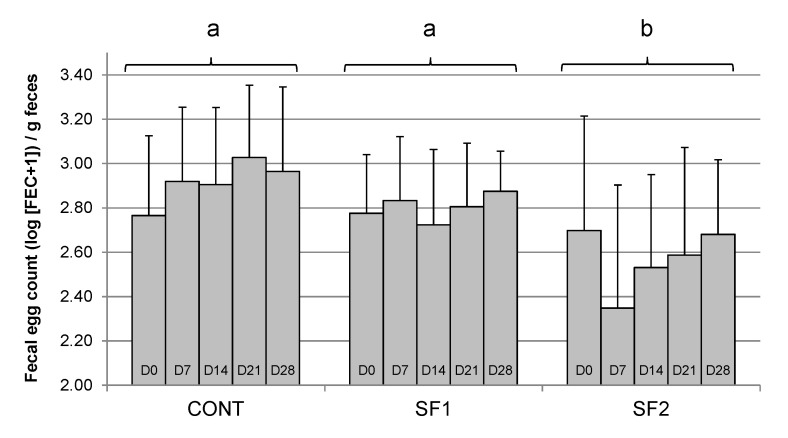
Changes in mean fecal egg count (log-transformed FEC) in naturally infected horses fed a control diet (CONT), a diet providing 1.53 g (SF1) or 3.06 g (SF2) DM pelleted sainfoin/kg BW/day from D1 to D28 during the pre-anthelmintic treatment period. Mean values ± SD are reported. a, b: different letters indicate significant differences between diets.

During Period 1, no evidence for Day × Diet interaction (*p* = 0.89) or temporal variation (Day effect, *p* = 0.80) was found (Figure 1). FEC was significantly reduced in horses fed the diet with the highest sainfoin content (SF2, 2.57 ± 0.45 log [FEC + 1]/g) compared to horses fed CONT (2.92 ± 0.33 log [FEC + 1]/g) diets or SF1 (2.80 ± 0.26 log [FEC + 1]/g) (*p* = 0.003) diets. A model run on percent change with respect to FEC at D0 (which accounts for any possible difference in initial values among horses) provided the same results, namely an effect of diet (*p* = 0.0257).

**Figure 2:**
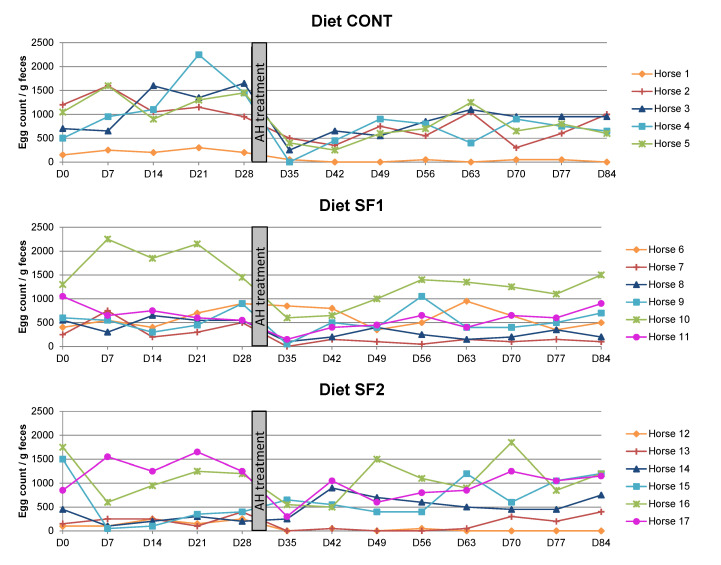
Individual changes in fecal egg count (FEC) in naturally infected horses fed a control diet (CONT), a diet providing 1.53 g (SF1) or 3.06 g (SF2) DM pelleted sainfoin/kg BW/day. Each line refers to an individual horse. FEC was assessed on a weekly basis from D0 to D84. After four weeks, all individuals were treated with an anthelmintic drug (AH treatment).

Plotting temporal changes in FEC for each individual horse showed substantial inter-individual variation (Figure 2). Using coefficients of variation of FEC as the dependent variables showed that, when receiving the diet with the highest sainfoin contents (SF2), inter-individual variation increased compared to the other experimental groups (mean ± SD: SF2, 0.18 ± 0.04; CONT, 0.12 ± 0.01; SF1, 0.10 ± 0.02) (*p* = 0.003). CV did not vary throughout the time (Day, *p* = 0.36).

### 3.3. Effect of Diet on FECRT

Fenbendazole had a very reduced efficacy, as shown by the low FECR values and the large uncertainty intervals (Day 7 post-treatment: 67.02% [UI = 45.9%/79.9%]; Day 14 post-treatment: 47.4% [UI = 24.4%/63.4%]). When using the individual reductions in FEC before and after fenbendazole treatment, we did not find any effect of diet, either at Day 7 (*p* = 0.424), or at Day 14 post-treatment (*p* = 0.269).

### 3.4. Effect of Diet on Fecal Egg Count during the Post-Anthelmintic Treatment Period (D35 to D84)

**Figure 3:**
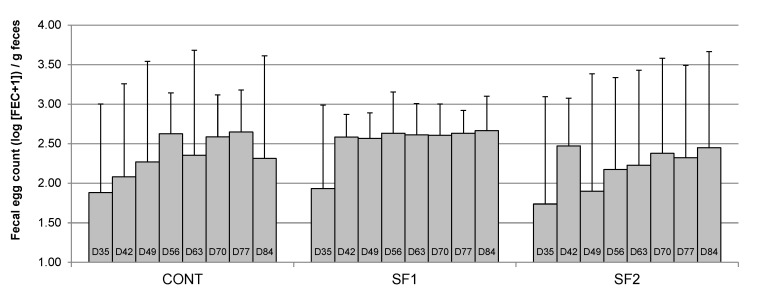
Changes in mean fecal egg count (log-transformed FEC) in naturally infected horses fed a control diet (CONT), a diet providing 1.53 g (SF1) or 3.06 g (SF2) DM pelleted sainfoin/kg BW/day during the post-anthelmintic treatment period. Mean values ± SD are reported.

During Period 2, we did not find any effect of Day (*p* = 0.48), Diet (*p* = 0.26) or their interaction (*p* = 0.99) on FEC (Figure 3). As for the pre-anthelmintic treatment period, Diet had an effect on the coefficients of variation of FEC (*p* = 0.0006). Horses fed the SF1 diet had significantly lower coefficients of variation (0.19 ± 0.14) than horses fed CONT diets (0.43 ± 0.19) or SF2 (0.55 ± 0.17). Coefficients of variation of FEC did not vary during Period 2 (Day effect, *p* = 0.07).

### 3.5. Effect of Diet on Larval Motility

Fifty-four samples (out of a total of 68 coprocultures) had more than three larvae per 100 µL and were included in the analysis of larval motility (mean ± SD: 26.2 ± 35.7 larvae/100 µL). No significant correlation was found between the number of larvae observed and the motility score (r = +0.09, *p* = 0.52).

**Figure 4:**
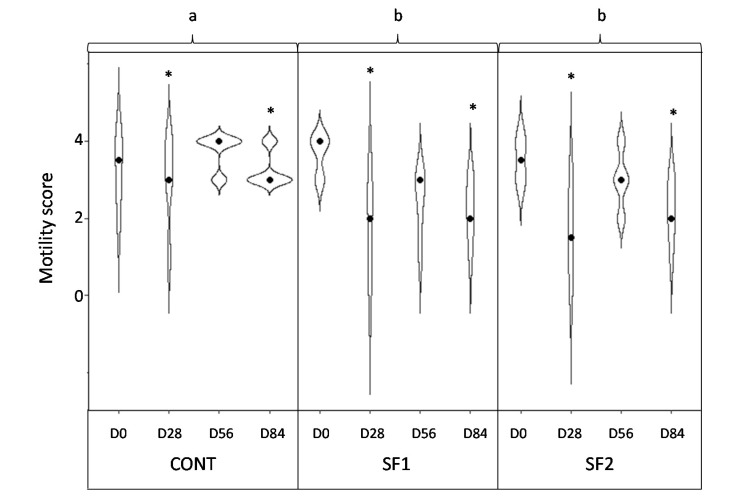
Violin plot representing the changes in larval motility score in naturally infected horses fed a control diet (CONT), a diet providing 1.53 g (SF1) or 3.06 g (SF2) DM pelleted sainfoin/kg BW/day (dots represent median values). a, b: different letters indicate significant differences between diets. * *p* < 0.05 compared to the motility at D0.

No significant interaction of Day × Diet was found in larval motility (*p* = 0.47; Figure 4). Larval motility was significantly higher at D0 compared to D28 and D84 (Day effect, *p* = 0.008). Larvae from horses fed sainfoin were less motile than larvae from control-fed horses (Diet effect, *p* = 0.04).

## 4. Discussion

Our findings showed that horses naturally infected with strongyles and fed with sainfoin had reduced parasite egg shedding, but this finding was restricted to the period prior to the anthelminthic treatment, and released larvae with lower motility compared to control-fed animals.

To date, only one in vivo study evaluating the effect of sainfoin has been conducted in horses naturally infected with cyathostomins [25]. This previous work did not report any effect of sainfoin on egg excretion, since FEC similarly declined in all horses during the 18 days of monitoring, despite a diet providing 70% DM sainfoin pellets. This achieved a threshold of condensed tannins of 3.6% in the ration, as recommended in ruminant studies [24]. We provided a smaller amount of sainfoin (0.22% and 0.44% condensed tannins for SF1 and SF2 on a DM basis, respectively), to meet the European nutritional recommendations for equines in terms of hay ingestion. These guidelines recommend that the minimal amount of daily forage intake should be 15 g DM/kg BW, to respect equine ethological needs and health considerations [27]. We followed this recommendation to fix the hay ingestion level, and we complemented the diet with wheat bran and/or sainfoin pellets to achieve 100% of energetic needs. Any possible effect of sainfoin, in terms of FEC reduction during the period that preceded the anthelmintic treatment, seemed therefore to arise despite the smaller amounts of tannins provided in the rations. This suggests that other phenolic compounds in sainfoin, such as some flavonols (narcissin, nicotiflorin, rutin, quercetin, luteolin), could also carry anthelmintic properties, as already shown in vitro [31,32] and suggested in vivo [33,34].

The possible anthelminthic properties of sainfoin might also involve other pathways not directly related to a nematocidal activity. These might involve both microbiota- or immune-mediated effects. Polyphenols are poorly absorbed in the small intestine of a monogastric animal, leading to a large proportion entering the hindgut, where they can directly modulate hindgut microbiota and/or the host immune system [35,36]. For instance, rats fed a diet containing polyphenol were less likely to suffer from dysbiosis under dietary stress, and had higher amounts of fecal mucin and immunoglobulin A [37]. Microbiota and the mucosal immune system interact with helminths, as demonstrated by several recent studies in mammals [38,39]. Thus, sainfoin could regulate strongyles through alteration of other pathways in the hindgut. Moreover, polyphenols can be broken down by the enzymatic activity of microbiota in the hindgut [40]. Considering the inter-individual variability in equine hindgut microbiota [41], the bioavailability and bioefficacy of polyphenols and their metabolites could vary tremendously among individuals [36,40]. To monitor the dynamics of egg excretion, we weekly assessed FEC in 17 horses. The FEC trajectories showed a substantial inter-individual variability, which is a common finding in nematode-infected animals [42]. However, sainfoin also increased the among-individual variation in FEC. Before the anthelminthic treatment, CV of FEC was significantly higher for horses in the SF2 group. Increased among-individual variability in infection dynamics in sainfoin-fed animals corroborates the hypothesis that sainfoin might interact with other host-intrinsic traits, such as the immune response and/or microbiota diversity and composition.

The reduction of egg excretion during the pre-anthelmintic treatment period corroborates the results reported in ruminants, namely a rapid reduction in FEC in naturally infected herbivores feeding on sainfoin [17,19,20]. Depending of the gastrointestinal segment of ruminants and the targeted nematode species, egg shedding reduction is either due to a decrease in the number of adult worms [13,16,21] or a lower female worm fertility (per capita fecundity) [13,15,18,33,34]. Since we did not use any invasive techniques, allowing us, for instance, to assess the number of adult worms, we could not establish the exact mechanism underlying the reduction in egg shedding.

We also found that sainfoin had an inhibitory effect on the motility of strongyle infective larvae. An in vitro experiment conducted on infective larvae of the three commonest nematodes of small ruminants showed that sainfoin could inhibit motility of abomasal *Haemonchus contortus* and intestinal *Trichostrongylus colubriformis,* but not of abomasal *Teladorsagia circumcincta* [43]. Using transmission electron microscopy to evaluate ultrastructural changes in ensheathed L3 of abomasal *Haemonchus contortus* and intestinal *Trichostrongylus colubriformis* after in vitro incubation with sainfoin extracts, Brunet et al. (2011) showed that the two nematode species presented lesions, such as alterations to the hypodermis, or degeneration of muscle cells [44]. This could explain the partial or total inhibition of larval motility after contact with sainfoin. In our study, L3 larvae developed in feces during coproculture, and we can speculate that the impairment of biological functions of larvae from sainfoin-fed horses might have been caused by residual polyphenol contents in the feces. In cattle [45] or sheep [46] feeding on sainfoin, the changes in condensed tannin concentrations were measured along the different digestive segments. Lower tannin concentrations were found in the digesta and feces compared to the original feeds, but concentrations remained similar all along the digestive tract until the feces. No changes in the structural features of assayable condensed tannins were observed. Thus, we can hypothesize that tannins maintain a similar pattern in horse digesta and feces. A reduction of infective larval motility due to sainfoin might have negative effects on the dynamics of infection in pasture, since larvae with low motility might be less successful in infecting the host. In addition, given that larval exsheathment has been shown to be delayed after incubation with sainfoin extract [47], even if infective larvae were ingested in pasture, their infection dynamics in the digestive tract might still be altered.

The anthelmintic treatment only produced a 67% (UI = 45.9%/79.9%) reduction of egg excretion by Day 7 post-treatment, suggesting widespread resistance to fenbendazole [29]. As fenbendazole had never been previously used in our farm, resistant strongyles probably originate from the horses’ former stable. Nevertheless, the diet did not affect the efficacy of the treatment, since FECRT did not vary among groups either at Day 7 or at Day 14 post-treatment. Overall, we did not find any effect of sainfoin on egg excretion during the period that followed the fenbendazole treatment. FEC increased similarly in the three groups of horses during this period. The lack of diet effects on FEC during the post-anthelmintic treatment period should be fully considered because it calls into question the possible efficacy of providing sainfoin as a strategy to mitigate strongyle infection in horses at any time. Given the high levels of resistance to the drug, it seems plausible to assume that only resistant worms were left following the treatment. One can wonder to what extent this might contribute to explaining the lack of diet effect of the fenbendazole treatment. Along the same lines, it is known that fenbendazole disrupts the horse’s hindgut microbiota structure, composition and function [7,8], as well as immune homeostasis [7]. If any anthelminthic properties of sainfoin depend on hindgut microbiota or immune function [48], sainfoin’s efficiency might have been affected in the post-fenbendazole period. These speculations about the possible reasons underlying the lack of diet effect during the post-fenbendazole treatment period do not mask the finding that, if sainfoin does have an anthelmintic effect, it appears to be context-dependent.

We found that naturally infected horses fed an SF2 diet showed a significant decrease in FEC during the pre-fenbendazole treatment period; however, the values were still, on average, higher (mean ± SD: 598 ± 556 EPG) than the threshold—often 200 EPG—commonly used to determine if an anthelminthic treatment is required [29]. This raises questions about the levels of egg excretion in healthy horses, and whether we should always aim to fully clear infections. The cutoff of 200 EPG (this can vary between 100 and 500 EPG depending on practitioners) appears to be related to traditional practices rather than being based on scientific evidence [49]. It was implemented for selective therapy, to treat only high spreader horses, with the aim of limiting the spread of anthelmintic resistance. However, this cutoff makes less sense in terms of equine health. First, although horses with less than 500 EPG have a lower worm burden, there is no linear correlation between FEC and worm burden [50]. Second, horses can harbor high worm counts without developing clinical disease, and there is no clear link between FEC and horse performance [51]. Finally, helminths have a long co-evolutionary history with their hosts and the microorganisms that live in the intestinal environment, and there is growing evidence suggesting that they might all contribute to maintaining intestinal homeostasis and regulating the host immune system [52,53]. Thus, an alternative strategy to a full eradication of nematode infection (which seems illusory) might be to maintain worm burden to levels that do not impair host health, while possibly providing benefits in terms of intestinal homeostasis maintenance. A steadiness in FEC could reflect the desired balance of the hindgut ecosystem. Further studies are definitely needed to investigate whether sainfoin could be a reliable natural way to regulate worm burden, while ensuring mutually beneficial relations between microbiota and host immunity.

## 5. Limitations

We acknowledge that our work has some limitations that we would like to address here. The study was based on the monitoring of FEC during a relatively short period of time before the anthelminthic treatment was applied. Further studies should therefore assess whether sainfoin given to infected horses might reduce FEC in the longer term. Additionally, our study was conducted during the spring, when young adult worms emerge from the mucosa after winter. Different results could have been obtained at a different time of year, at a different parasite developmental stage [42]. A larger sample size in the horse population would have given us a better statistical power to detect the interactions between treatment and time. Similarly, evaluation of mobility on a larger larva sample would have been more robust, particularly as that method is based on a subjective assessment. The use of a more effective anthelmintic drug would have allowed us to better assess whether the diets affected the rate of reinfection. Finally, using a larger range of sainfoin doses would have been useful to better appraise possible dose-dependent anthelmintic effects.

## 6. Conclusions

We found that horses fed with 3 g DM/kg BW of sainfoin excreted fewer parasite eggs prior to the anthelminthic treatment. Moreover, the high inter-individual variation in fecal egg count that we measured in sainfoin-fed horses suggests that anthelmintic activity might be modulated by intrinsic characteristics of the host, such as the immune response and/or the diversity or composition of the microbiota. The lack of effect of diet on FEC following the fenbendazole treatment also suggests that any possible anthelmintic properties of sainfoin might be context-dependent. Finally, during the whole study, sainfoin demonstrated an inhibitory effect on the motility of strongyle infective larvae, which suggests that it might carry the ability to disturb strongyle at different stages of the lifecycle.

## Figures and Tables

**Table 1:** Means ± SD of body weight, age and FEC at Day 0 for the three experimental groups. The *p*-values of one-way ANOVA testing if these initial values differed among groups were also reported.

Variable	Experimental Groups			*p*-Value
	CONT	SF1	SF2	
Body weight (kg)	530 ± 23	520 ± 35	528 ± 44	0.885
Age (yrs)	7 ± 2	8 ± 2	6 ± 1	0.145
FEC (number of eggs/g of feces)	720 ± 422	692 ± 402	800 ± 697	0.936

**Table 2:** Composition of sainfoin and experimental diets.

Ingredients (% DM Intake)	Sainfoin	Experimental Diets		
		CONT	SF1	SF2
Hay		83.0	83.0	83.0
Wheat bran		17.0	8.5	0.0
Sainfoin		0.0	8.5	17.0
Biochemical composition				
DM (%)	88.1	92.6	92.5	92.4
DE (kcal/kg DM)	2550	2272	2253	2235
Crude protein *	16.6	8.75	8.69	8.63
NDF *	39.9	57.4	57.4	57.3
ADF *	31.6	33.5	35.0	36.6
ADL *	7.6	4.27	4.56	4.86
Starch *	0.8	3.31	1.77	0.22
Sugars *	8.9	8.44	8.67	8.90
Crude fat *	2.5	2.11	1.89	1.67
Ash *	8.4	6.25	6.37	6.49
Calcium *	1.56	0.44	0.57	0.69
Phosphorus *	0.29	0.39	0.32	0.24
Condensed tannins *	2.6	0.00	0.22	0.44

* Proportions, values were expressed on a dry matter basis. CONT, diet distributed to the control group; SF1, diet distributed to the sainfoin 1 group; SF2, diet distributed to the sainfoin 2 group.

## Data Availability

Not applicable.
